# The relationship between chronic perceived stress and error processing: evidence from event-related potentials

**DOI:** 10.1038/s41598-019-48179-0

**Published:** 2019-08-12

**Authors:** Jianhui Wu, Mengjiao Feng, Yutong Liu, Huihua Fang, Hongxia Duan

**Affiliations:** Center for Brain Disorder and Cognitive Science, Shenzhen Key Laboratory of Affective and Social Cognitive Science, Shenzhen University, Shenzhen, 518060 China

**Keywords:** Cognitive neuroscience, Human behaviour

## Abstract

Prolonged exposure to stress has a wide effect on the brain and cognition. Error processing, as one of the crucial components of executive function, plays an important role in cognitive and behavior control. However, to date, there is little research addressing the relationship between chronic perceived stress and error processing. The present study aims to explore the relationship between chronic perceived stress by the Cohen Perceived Stress Scale (PSS) and different stages of error processing by the method of Event-Related Potential (ERP). The error processing was tested in a classical auditory Go/NoGo paradigm, and ERP components including early Error-related Negativity (ERN) and late Error Positivity (Pe) were computed as the indices of error processing. The results showed that the PSS score was positively correlated with the Pe amplitude but not with the ERN amplitude. The correlation between PSS and the Pe amplitude holds true even after controlling the trait anxiety and depression symptoms. These results suggest that the higher the chronic stress level, the more sensitive the individuals are to their own errors as well as the more emotional/motivated attention the individuals distributed to their own errors.

## Introduction

Inevitably, we will encounter various stressful events in our daily life. Acute and moderate stress may cause adaptive stress response and allocate limited cognitive resources to the brain regions and organs that are currently demanded, thus increasing vigilance in response to changes and threats in the environment^1,2^. However, longstanding animal and human studies have shown that prolonged exposure to stress may have a detrimental influence on mental and physical health^3,4^. In healthy individuals, chronic stress impairs prefrontal-dependent executive function such as working memory^5^, attentional control^6^ and decision making^7^, while enhances amygdala-dependent emotional processing such as exaggerated emotional response^8^, emotional learning^9^ and aggressive behavior^10^.

The ability to monitor one’s own behavior is another crucial executive function implemented by medial prefrontal cortex (mPFC) and anterior cingulate cortex (ACC), and it includes multiple dynamic stages such as continuous examination of ongoing behavior, augmented attention and alertness after mistake commission and mobilization of cognitive control to reduce the probability of committing more errors^11–14^. Error is often considered a potential threat and may activate defensive motivation systems to protect the organism. Detection of errors can improve future performance in general, while it has a survival significance in the environment full of threat and stress^15,16^.

The dynamic stages of error processing can be examined by event-related potentials (ERPs), and two specific response-locked components are the error-related negativity (ERN or Ne) and the error positivity (Pe). The ERN is a negative ERP waveform, appearing at the frontocentral region between 0 and 100 ms after the onset of the error commission, and the magnitude of ERN might reflect the automatic detection of error or the mismatch between actual response and required response^17,18^. Following the ERN, the Pe is a positive ERP waveform, emerging at the centroparietal region between 200 to 400 ms after the onset of erroneous response. Studies indicate that the Pe reflects the conscious recognition of the error^19^ or the motivational/emotional evaluation^18^.

Several clinical studies have found abnormal error processing at both stages in various psychiatric disorders. Patients with internalized disorders such as compulsive-obsessive disorder, anxiety and depression were sensitive to errors, revealing as a larger ERN amplitude than healthy controls^20–22^; while individuals with externalized behaviors like antisocial behavior, substance abuse and aggressive personality had impaired behavior monitoring and a smaller ERN amplitude than healthy individuals^23,24^. Therefore, some researchers suggested the ERN as an endophenotype for psychopathology^25,26^. Similarly, studies with healthy individuals revealed that the magnitude of ERN amplitude is positively related to trait anxiety^27^, trait negative affect^28^ and trait behavioral inhibition^29^, and negatively related to impulsivity^30^. Unlike the ERN’s proneness to trait characteristic, the Pe seems to be influenced a lot by the individual’s state. The Pe was amplified when participants were over concerned about errors^31–33^. Moser and colleagues (2005) found a smaller Pe amplitude but equal ERN under threat condition (with the presence of a Chilean rose-haired tarantula) compared to the safe condition^34^. Another study with major academic examination as chronic stressor again found that exam group had a larger Pe amplitude but similar ERN than non-exam group^35^.

Measurements of chronic stress on human in recent studies have been heterogeneous and included different definitions such as academic examination^35,36^, socioeconomic stress^37^ or reporting negative life events^38^. However, it has been proposed that it might be that “perceived stress”, the general perception about how individuals find environmental demands exceed their capacity regardless of the type of the environmental demand, is linked with long-term stress response and physical disease^39,40^. Orem *et al*. (2007) found that the higher level of self-chronic perceived stress is associated with worse (longer reaction time) set-shifting performance^41^. Furthermore, on the brain structural level, a longitudinal study revealed that chronic stress measured by Perceived Stress Scale (PSS) over an approximate 20-year period predicted decreased grey matter volume in the prefrontal cortex and hippocampus, even after controlling several potentially confounding factors^42^. Functionally, Treadway and his colleagues (2013) also found that higher perceived stress by PSS was associated with blunted activation of medial prefrontal cortex following behavioral feedback^43^. In line with these results, it is possible that perceived stress is also associated with disrupted prefrontal-dependent error processing.

Therefore, to test the possibility, the aim of the present study was to investigate the continuous spectrum of chronic perceived stress levels, as opposed to categorized groups, in order to explore its relationship with error processing at neural level by ERP method. To address this question, chronic perceived stress was assessed by the PSS (10 item version)^44^, a widely-used instrument to measure the perceived predictability, controllability and overload of daily life over the past one month period. The error processing was evaluated by the classical Go/NoGo task. The Go/NoGo task is a well-established measure for both correct and failed inhibitory control across neuroimaging and ERP studies^45,46^. Given the evidence reviewed above that chronic stress is associated with diminished brain function on behavioral, structural and functional levels, we hypothesized that chronic perceived stress is related with abnormal error processing.

## Results

### Subjective measurements

The mean PSS10 score was 16.08 (SD: 5.36). The mean CES-D score was 11.52 (SD: 8.317). The mean trait anxiety score was 42.68 (SD: 7.36).

### Behavioral performance

The mean omission error rate to Go trials was 8.86% (SD: 8.95), and participants responded with an average RT of 403.58 ms (SD: 66.39 ms). Commission errors occurred on 25.17% of NoGo stimuli with an average of 30.2 (SD: 19.48) false alarms to NoGo trials per participant.

### ERP results

**Figure 1:**
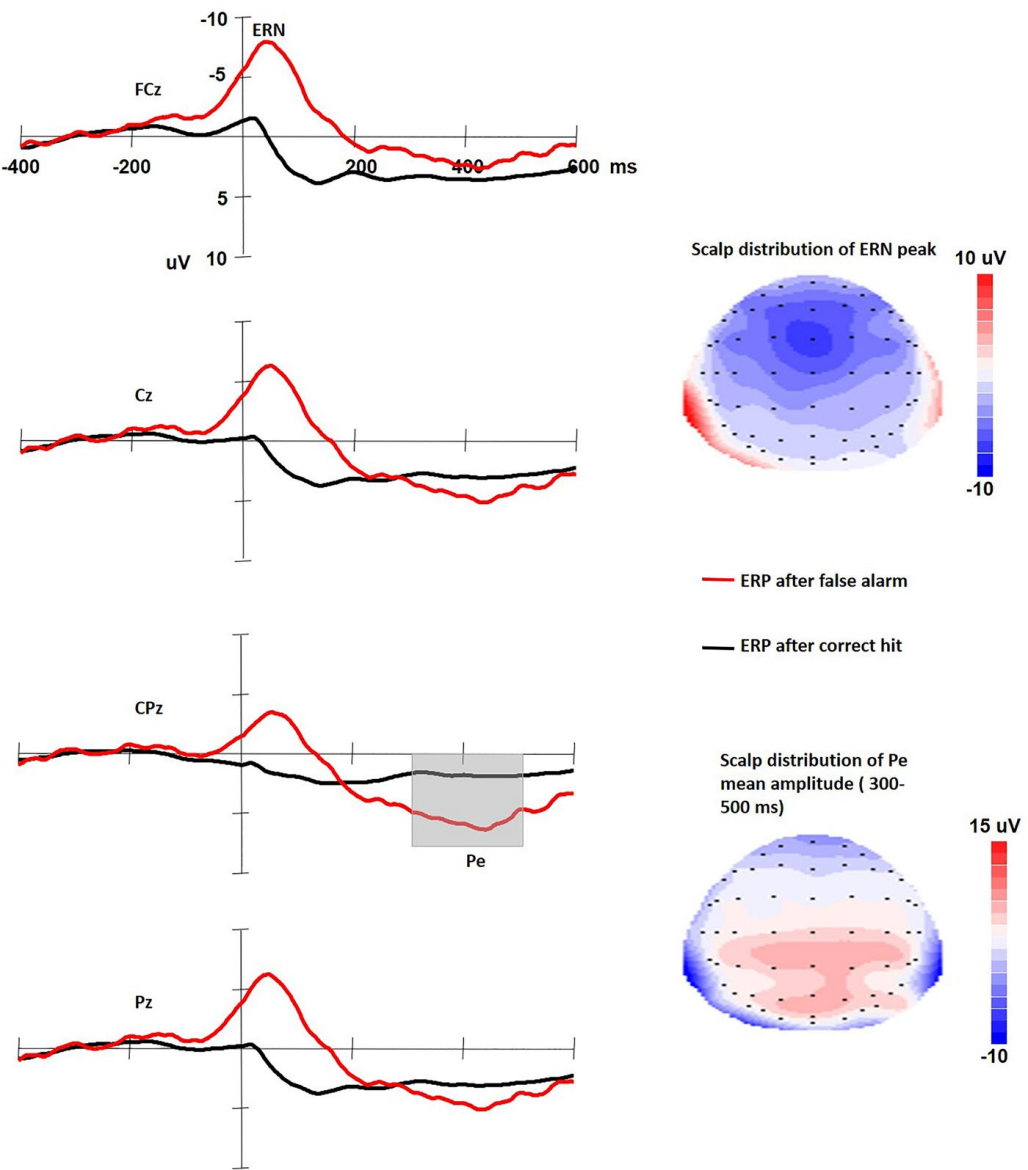
Left: The ERP waveforms time locked to both correct hit response to Go stimuli and false alarm response to NoGo stimuli. Right: The scalp distribution of the ERN and the Pe.

Figure 1 shows the ERP waveforms time locked to both correct hit response to Go stimuli and false alarm response to NoGo stimuli. Results from the repeated measurement ANOVAs with Greenhouse-Geisser adjusted *p*-values showed that the ERN amplitude to erroneous response is larger than to correct response (−9.607 μV (SD: 5.20) vs. 0.499 μV (SD: 2.108), F(1,57) = 217.504, *p* < 0.001). The mean latency of the ERN was 48.14 ms (SD: 33.40). The topographic distribution for the peak ERN (see Fig. 1 up-right) is mainly centered at midline frontocentral site. Results from the repeated measurement ANOVAs showed that the Pe amplitude to erroneous response is larger than to correct response (5.10 μV (SD: 5.06) vs. 1.214 μV (SD: 1.707), F(1,57) = 34.738, *p* < 0.001). The topographic distribution for the Pe (see Fig. 1 down-right) is more posterior than the ERN, and mainly around centroparietal region. The scalp distribution patterns of the ERN and the Pe were consistent with previous studies^46^.

### Correlational analysis

There was a significantly positive relationship between PSS10 and the Pe amplitude (rho = 0.288, *p* = 0.029). No significant associations were found between CES-D or trait anxiety score and the Pe amplitude (rho = 0.077 and 0.178 respectively, *p*_*s*_ > 0.10). PSS10, CES-D or trait anxiety scores were not associated with the ERN amplitude and latency (|rho|_s_ = 0.006~0.150, *p*_*s*_ > 0.10). PSS10 was marginally positively correlated with false alarm rate (rho = 0.233, *p* = 0.07). CES-D or trait anxiety scores were not associated with the hit rate and RT (rho_s_ = −0.102 and −0.131, respectively, *p*_*s*_ > 0.10).

**Figure 2:**
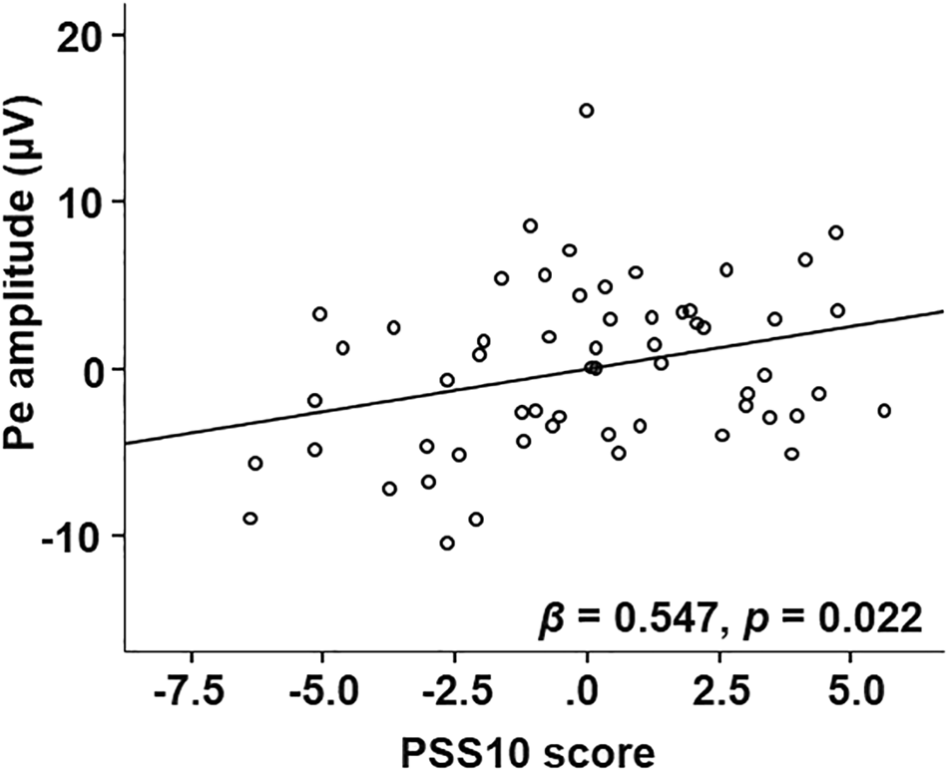
The scatterplot of the relationship between the PSS10 score and the average Pe amplitude.

Then, hierarchical regression analysis was performed on the Pe amplitude, including the variables CES-D and trait anxiety at the first step and PSS10 at the second step. The results of the regression are summarized in Table 1. The chronic perceived stress (PSS10) accounted for 7.9% of the variance in the Pe amplitude (△R^2^ = 0.079, F(3,57) = 2.619, *p* = 0.06) above-and-beyond all the following step 1 covariates. Only PSS10 significantly predicted the Pe amplitude (*β* = 0.547, *p* = 0.022, see Table 1 and Fig. 2).

**Table 1:** Stepwise regression analysis on Pe amplitude.

Model	Predictor	B	β	t	p	△R^2^	R^2^	F	p
Step 1							0.038	1.08	0.347
	Trait anxiety	0.091	0.134	0.643	0.523				
	CES-D	0.043	0.076	0.341	0.734				
Step 2						0.079	0.127	2.619	0.06
	Trait anxiety	−0.053	−0.077	−0.351	0.727				
	CES-D	−0.123	−0.203	−0.877	0.385				
	PSS10	0.508	0.547	2.35	0.022

## Discussion

The present study investigated the relationship between chronic perceived stress, measured by a widely used and validated PSS10, and error processing by ERP method in healthy young males. The result showed that chronic perceived stress predicted larger Pe amplitude even after controlling depression symptoms and trait anxiety.

Chronic perceived stress positively predicted the late stage of error processing rather than the early stage, i.e., the higher the level of chronic perceived stress, the larger the Pe amplitude. This result was consistent with the result from Wu *et al*. (2014), in which they showed that the Pe amplitude was larger while the ERN did not differ compared the exam group who went through a long-term preparation for an important academic examination to the control group^35^. However, many factors like self-perceived overall competence and motivation^47^ may differ between the exam group and control group. Furthermore, the authors also found that the self-reported importance of the academic examination was significantly positively correlated with the Pe amplitude^35^. Therefore, it cannot be ruled out that the increased Pe amplitude in the exam group they found is due to the difference in academic motivation between the two groups rather than the chronic stress level. The present study, however, by using the perceived stress scale excluded the response variability within human individuals to the same stressor, for example, preparing for an academic examination. Humans appraise the uncontrollability, unpredictability and overload of their life by higher-order neocortical processing, which is proposed to activate the hypothalamic-pituitary-adrenal axis and stress response^39,40,48,49^. Moreover, by using continuous variables rather than categorical method improved statistical power and reduced parameter estimation bias^50^. In addition, the positive associations between chronic perceived stress and Pe amplitude were replicated after controlling depression and trait anxiety within prediction models. These results provided direct neurophysiological evidence for the association between perceived stress and error processing in a non-clinical sample of young males.

According to the previous literature, the Pe might reflect conscious recognition of errors and the level of subjective motivation and emotional assessment of errors^18^. The increase in Pe amplitude with higher chronic perceived stress may indicate that the higher of chronic perceived stress, the more an individual is aware of the errors they made and in turn, the more emotional/motivational attentional resource they will allocate to the errors. Since error is considered as potential threat^15,16^, this interpretation echoed with the proposal that individuals under chronic stress are more sensitive to negative stimuli^51^. High perceived stress has been found linked to physical health like a higher risk of cardiovascular disease, hypertension as well as shorter telomere length^39,40,52^. Wirkner and colleagues (2019) found that higher chronic stress level measured by hair cortisol level was associated with enhanced emotional arousal toward affective stimuli as indicated by late positive potential^53^. Furthermore, imaging studies have consistently revealed that brain regions that process attention, executive control and emotion are influenced by chronic perceived stress. For example, Lederbogen and colleagues (2011) found that compared to the non-urban citizens, urban citizens who had higher chronic stress level showed larger amygdala response when confronted with acute stress^54^. Higher self-reported chronic stress was associated with lower activation of the ACC as well as the lower connectivity between amygdala and ACC during odor perception^55^. On the structural level, higher self-reported chronic stress was associated with smaller volume in mPFC and ACC in a cross-sectional study^56^, and even predicted decreased volume in orbitofrontal cortex in a longitudinal study^42^. Considering the general regulatory role of ACC in behavioral monitoring and emotion^17,57^, the positive correlation between chronic perceived stress and the Pe amplitude in the current study might be due to the chronic stress-induced excessive activation of the amygdala with reduced ability of the ACC to regulate the overactivated amygdala, leading to exaggerated emotional assessment of the errors. Interestingly, the level of chronic perceived stress was related with the Pe amplitude rather than the ERN component, suggesting chronic stress has a distinct influence on different stages of error processing. Previous studies have suggested that the ERN may reflect stable factors. For example, the ERN amplitude was positively correlated with trait anxiey^27^, trait negative affect^28^ and behavioral inhibition^29^ and negatively associated with impulsivity^30^. The result of the genetic study from Fallgatter *et al*. (2004) to some extent supported this hypothesis, in which they found that the ERN but not Pe amplitude was associated with the short allele of the serotonin transporter. Correspondingly, several studies found that the Pe component is more inclined to be affected by environmental factors and psychological states^31,33–35^. The current study provides more evidence that chronic stress is associated with the late stage (i.e., Pe) rather than the early state (i.e., ERN) of error processing. Future research might be better able to address this distinction by twin studies.

Our results may have some implications. The relationship between chronic perceived stress and error monitoring suggests that individuals perceived more chronic stress might be more sensitive to negative events such as committing an error. This increased sensitivity to errors, on the one hand, can promote behavioral adjustment to avoid making more errors in the future^58^. On the other hand, oversensitive to mistakes might also constitute a risk factor for mental illness such as mood and anxiety disorders^24,25^. Epidemiological studies showed that individuals who perceive negative experiences to be uncontrollable and overwhelming are more likely to develop depression after stressful traumatic events^59,60^. The link between higher perceived stress and psychiatric symptoms might partially be mediated by oversensitivity to the errors they made in daily life. Therefore, our results highlight the potential therapeutic value for stress-reduction interventions by adjusting individual’s error evaluation process.

There are some limitations need to be addressed in the current study. First, the sample of this study was male university students. Therefore, the results we obtained here might not be able to generalize to other groups. Secondly, the chronic stress level was assessed by a subjective perceived stress scale without physiological indicators such as hair cortisol. However, it is worthwhile to mention that people might appraise the impact and severity of the similar negative life events differently^61^, and it was proposed that it is the stress appraisal and coping causes long-term strain and finally lead to psychological and physiological disease^62^.

In summary, the present study help identify how variation in perceived chronic stress influence neural response involved in error processing. Understanding the relationship between perceived chronic stress load and error processing in healthy people without the contamination of psychiatric-related factors is important to understand stress-related psychopathology. Our findings revealed that chronic perceived stress was associated with the later stage rather than the early stage of error processing, suggesting that the higher level of chronic perceived stress, the more an individual is aware of the errors they made and in turn, the more emotional/motivational attentional resource they will allocate to the errors.

## Method

### Participants

This study is part of a larger project addressing the relationship between cognitive function and stress response. Due to the well-documented sex differences in stress response^63^, only male students were recruited through advertisements in a local university. Prescreening excluded people with acute or chronic psychiatric, physiological or neurological disorders. Sixty male students with normal auditory ability participated in the experiment. All participants were medication free and without smoking or drinking habit. The average age of the participants was 21.15 years (standard deviation (SD): 0.83, range: 20~23 years) and the average education year was 13.55 (SD: 0.70, range: 13~16 years).

### Task and procedure

At arrival, participants took a rest and filled out questionnaires on the computer. Subsequently, participants were seated on a comfortable chair in a normally illuminated room to perform a classical auditory Go/NoGo task^45,46^ while EEG was recorded. For the auditory Go/NoGo task, the targets (Go stimuli) were 1000 Hz pure tones and the non-targets (NoGo stimuli) were 1032 Hz pure tones (10 ms rise and fall times). The sound pressure was adjusted to a comfortable level. All the tones were presented binaurally over headphones. After an initial practice block of 10 trials, two experimental blocks each including 310 trails (20% NoGo and 80% Go probability) were completed with 2 min breaks between blocks. The duration of the tones was 200 ms and the stimulus-onset asynchrony randomized from 900 to 1100 ms. During each trial, one of the two tones were presented. Participants were instructed to respond as fast as possible by pressing a button on the keyboard once they heard the Go stimuli. The sequence of Go and NoGo stimuli was pseudorandom thus the consecutive presentation of two NoGo stimuli was avoided.

### Questionnaires

Chronic perceived stress was assessed by the 10-item PSS (PSS10)^44^. This standardized instrument evaluates how much an individual perceives life as unpredictable, uncontrollable and overloading, which are the core components of the stressful experience and physiological stress response^48,49^. The scale measures perceived stress over the last one month on a 4-point Likert scale ranging from 0 (never) to 4 (always).

Depressive symptoms were assessed by the Center for Epidemiologic Studies Depression Scale (CES-D)^64^. Respondents indicated the extent of the symptoms they had experienced in the past week on a 4-point scale (0 = rarely or none of the time, 3 = most or all of the time).

Trait anxiety was also measured by the trait subscale from the State-Trait Anxiety Inventory (STAI)^65^.

CES-D and trait anxiety were used as covariates in our data analysis to correct for their potential relationship with perceived stress and error processing^20–22^.

### EEG recording and preprocessing

The EEG was recorded with Ag/AgCl electrodes from 64 scalp sites mounted in an elastic cap (Neuroscan Inc., Charlotte, North Carolina, USA) according to the international 10–20 system, with online reference to the left mastoid. Data were re-referenced offline to the average of both mastoids. One pair of electrodes placed above and below the left eye was used to record vertical eye movement and another pair placed 10 mm from the outer canthi of each eye to monitor horizontal eye movement. The impedance from all electrodes was below 5 KΩ. Signals were amplified with bandpass filter at 0.05–100 Hz and digitized at 1000 Hz.

The EEG data was processed by Scan 4.3 software (Neuroscan, USA). The data was further filtered with a 30 Hz lowpass filter. Ocular artifacts were removed from the EEG signal by a regression procedure implemented in the Neuroscan software. Trials with artifacts were automatically rejected with a criterion of ±100 μV. EEG data were epoched into periods of 1000 ms, including 400–200 ms before response as baseline according to previous studies^35,66^, and time-locked to the onset of button press.

### Data analysis

For the behavior data, reaction time (RT) of the correct response to the Go trials was measured relative to stimulus onset. There were two types of accuracy evaluated: omission errors which calculated as the percentage that the participants did not respond to the Go trials and commission errors (or false alarm rate) which defined as the percentage of erroneous responses to the NoGo trials. Trials with RT below 50 ms and above 1000 ms were excluded.

For the EEG data, only error responses of the NoGo trials (false alarm) were averaged for the ERN and Pe. According to our own data (see Fig. 1) and previous researches^35,46^, the peak amplitude and latency of the ERN at FCz and Cz sites where the ERN has its peak was detected 50 ms before and 150 ms after a false alarm response, and the mean amplitude of the Pe at CPz and Pz sites was measured between 300 and 500 ms after a false alarm response. To reduce statistical error, the average ERN from FCz and Cz and the average Pe from CPz and Pz were calculated to conduct the following correlational analysis.

Due to the non-normal distribution of the ERP data, nonparametric Spearman correlations were performed to assess relationships between ERP indices of error processing (ERN amplitude and latency, Pe amplitude) and scores of psychological scales. In subsequent 2-step hierarchical regression analyses, we tested whether chronic perceived stress predicted the error processing independently of additional confounding factors. For these 2-step regressions, the error processing index which had a significant relationship with psychological measurements was entered as dependent variable. In step 1, we entered trait anxiety and depressive symptoms; in step 2, we entered the PSS10 (our indicator of chronic perceived stress). The unique percentage of variance of error processing explained by chronic perceived stress was calculated by the change in R^2^ (ΔR^2^) in step 2. All the statistics were conducted by SPSS23.0, and two-tailed tests with the threshold for significance was set to *p* < 0.05.

### Ethics

All participants gave written informed consent and were paid for their participation. This experiment was approved by the Ethics Committee of Human Experimentation in the medical department at Shenzhen University. The experiment was conducted in accordance with relevant guidelines and regulation.

## Data Availability

The anonymous behavioral and EEG data will be made available for research purposes upon requests.
